# Broccoli Sprouts and Their Influence on Thyroid Function in Different In Vitro and In Vivo Models

**DOI:** 10.3390/plants11202750

**Published:** 2022-10-18

**Authors:** Paweł Paśko, Paweł Zagrodzki, Krzysztof Okoń, Ewelina Prochownik, Mirosław Krośniak, Agnieszka Galanty

**Affiliations:** 1 Department of Food Chemistry and Nutrition, Jagiellonian University Medical College, Medyczna 9, 30-688 Kraków, Poland; 2 Department of Pathomorphology, Jagiellonian University Medical College, Grzegórzecka 16, 31-531 Kraków, Poland; 3 Department of Pharmacognosy, Jagiellonian University Medical College, Medyczna 9, 30-688 Kraków, Poland

**Keywords:** broccoli sprouts, thyroid, cancer, hypothyroidism, rats

## Abstract

Broccoli sprouts are a super vegetable; however, they have possible negative effects on thyroid function, which is especially important for patients with hypothyroidism. As the data on this issue are scarce, this study aimed to determine the safety and possible beneficial effect of broccoli sprouts both in vitro and in vivo. The in vitro model comprised the evaluation of the impact of broccoli sprouts on normal and neoplastic thyroid cells and the determination of their anti-inflammatory and antioxidant (IL-6, TNF-alpha, NO, and SOD) potential in macrophages. The in vivo model concerned the histopathological analysis of thyroid glands in healthy rats and rats with hypothyroidism (induced by iodine deficiency or sulfadimethoxine ingestion) fed with broccoli sprouts. The results of our study indicated that broccoli sprouts decreased the viability of thyroid cancer cells and prevented inflammation. The results also confirmed the satisfactory safety profile of the sprouts, both in vitro and in vivo; however, a further in-depth evaluation of this problem is still needed. Information on the influence of brassica vegetables on thyroid function is of great importance in terms of public health, particularly when taking into account that the risk of iodine deficiency, hypothyroidism, and thyroid cancer in the global population is still increasing.

## 1. Introduction

Broccoli (*Brassica oleracea* L. convar. *botrytis* var. *cymosa*) is recognized as one of the most important vegetables due to its nutritional value and chemoprotective potential [1]. It belongs to the family *Brassicaceae*, and the biological activity of this vegetable is mostly related to its sulfur compounds and polyphenol content [2]. Generally, the regular consumption of brassica vegetables may decrease the risk of breast, colorectal, bladder, lung, and prostate cancers [3]. Today, many different broccoli products, including florets, sprouts, stems, flour, flakes, powder, or crisps, are available in grocery stores and may be used to enrich one’s daily diet [2]. Despite being considered a super vegetable, broccoli is not devoid of a possible negative effect; this cannot be neglected, especially in the case of patients with hypothyroidism [4]. Glucosinolates and their derivatives, such as isothiocyanates and thiocyanates, which are present in broccoli in high amounts, are potentially responsible for this negative effect on thyroid function via the inhibition of the sodium/iodide symporter in the basolateral membrane of the thyroid follicle and thyroid peroxidase activity. These proteins are linked with iodide uptake and its oxidization in thyroid tissue, respectively [5]. In one study, the anti-thyroid potential of broccoli florets was rated as low due to it having the lowest level of progoitrin in comparison to other brassica vegetables (rutabaga roots, Siberian kale, or collards) [6]. Unlike broccoli florets, knowledge about the influence of broccoli sprouts on the thyroid is scarce. Presently, two in vivo studies about the influence of broccoli sprouts on the biochemical, immunological, and thyroid function of rats with hypothyroidism have been published [7,8], in addition to two human trials [9,10]. The animal studies showed that the addition of broccoli sprouts to the rats’ diet did not cause any significant changes in the levels of TSH, fT3, and fT4. Additionally, a protective effect of these sprouts against sulfadimethoxine-induced thyroid damage was noted. In rats with hypothyroidism, the sprouts exerted a beneficial influence on the antioxidant status of the thyroid gland and decreased IL-6 levels [7].

The first human study by Shapiro et al. [9] examining TSH, T3, and T4 levels did not show any significant changes in these parameters or even consistent thyroidal toxicities when broccoli sprouts were included in the diet of 12 healthy volunteers. Three different diet schedules were evaluated, where the amount of the sprouts was related to: (1) 25 μmoL isothiocyanate; (2) 25 μmoL glucosinolate; or (3) 100 μmoL glucosinolate per day. The sprouts were consumed three times per day for 7 days. In the second study, 45 females received a beverage containing broccoli sprouts (40 μmoL sulforaphane and 600 μmoL of glucoraphanin) for 84 days. The effect of this beverage on thyroid function and thyroid autoimmunity was not significant: no changes in TSH, fT4, and thyroglobulin levels were noted [10].

Because of the limited information on the influence of broccoli sprouts on thyroid function and their increasing presence in our daily diet, we decided to focus on this problem. To do this, different in vitro and in vivo models were applied to determine the safety and possible beneficial effect of broccoli sprouts. The in vitro model involved an evaluation of the impact of lyophilized broccoli sprout extract (BSE) on normal and neoplastic thyroid cells and the determination of their anti-inflammatory and antioxidant (IL-6, TNF-alpha, NO, and SOD) potential in macrophages. The in vivo model concerned the histopathological analysis of thyroid glands, including papillary formation, aggregates of lymphocytes, vacuole score, the quality of epithelial cells, follicular epithelial area, follicular luminal area, and overall thyroid area in healthy rats and rats with hypothyroidism (induced by iodine deficiency or sulfadimethoxine ingestion) fed with broccoli sprouts. The extract used in the present study was chemically profiled in our previously published papers [2,7]; the obtained data were used to reveal potential correlations with the results of the present in vitro and in vivo experiments by means of advanced chemometric methods.

## 2. Results and Discussion

### 2.1. Cytotoxic Acivity of Broccoli Sprouts on Thyroid Cells

**Figure 1:**
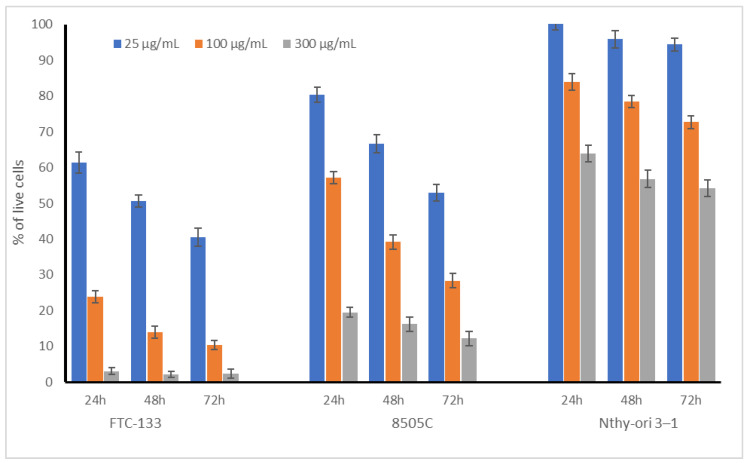
Cytotoxic effect of broccoli sprout extract (BSE) on cancer (FTC-133, 8505C) and non-neoplastic (Nthy-ori 3-1) thyroid cells. Cells were treated with 25, 100, and 300 µg/mL of sprout extract for 24 h, 48 h, and 72 h. Values are presented as the mean ± SD (standard deviation) (n = 3).

The experiment started with a preliminary in vitro evaluation of the toxic potential of broccoli sprout extract towards normal, non-neoplastic thyroid follicular epithelial cells (Nthy-ori 3-1). In addition, two thyroid carcinomas, FTC-133 and 8505C, differing in their metastatic potential (see Section 3.3.1 for details) were also included in the study to verify if BSE could possibly be useful in decreasing the development of thyroid cancer. The tested broccoli sprout extract varied in its influence on the examined cell lines; however, in all cases, the effect was dose dependent. The results are presented in Table 1 as IC_50_ values and in Figure 1 for three selected concentrations (0.025, 0.1, and 0.3 mg/mL). It should be strongly noted that the broccoli sprout extracts were not toxic to non-neoplastic thyroid (Nthy-ori 3-1) cells in the tested concentration range, even after prolonged exposure up to 72 h (Figure 1); the IC_50_ values were above C_max_ (Table 1).

At the highest tested concentration, more than 60% of Nthy-ori 3-1 cells were alive after 24 h, while their viability decreased after 48 and 72 h to 56 and 54%, respectively. However, such high concentrations could be treated as an acute toxic dose, which is impossible to achieve in the daily diet. The most important observation from our study was that the BSE was almost completely non-toxic to non-neoplastic cells at the concentrations at which significant and high cytotoxicity toward thyroid cancer cells was noted. Moreover, the cytotoxic effect of BSE significantly differed for FTC-133 and 8505C (IC_50_ 35.2 vs. 114.1 µg/mL for 24 h incubation) cells. More metastatic FTC-133 cells showed an increased response to the tested extract, especially at lower concentrations, in comparison to the undifferentiated carcinoma 8505C cells. As cancer cells within tumors may differ in terms of their proliferation rate and metastatic ability [11], these varied—but strong—effects of broccoli sprout extract on the different thyroid cancer cell lines used in the study is of great importance.

To the best of our knowledge, this is the first study to describe the influence of broccoli sprout extract on the viability of thyroid cells. Interestingly, in our previous study, kale sprouts (up to 500 μg/mL) did not have any significant cytotoxic effect on normal and cancer thyroid cells (8305C, FTC133) [12]. Wang et al. [13] evaluated the effect of sulforaphane on six different human thyroid cancer cell lines (8305C, IHH4, FTC133, BCPAP, TPC1, and K1) and noted that this compound significantly inhibited the proliferation of thyroid cancer cells in a dose- and time-dependent manner. Another study described the enhancement effect of the compound, combined with a photosensitiser (Photofrin), on anaplastic thyroid carcinoma FRO cells in photodynamic therapy. This combination resulted in cell cycle arrest via ROS generation and MMP depolarization, with the subsequent suppression of the expressions of the Ras, MEK, ERK, B-Raf proteins [14]. Sulforaphane is known to have significant cytotoxic properties in many different cell lines; the different mechanisms of its activity, such as cell cycle arrest in the G2/M and G1 phase or the blockage of phase I metabolic enzymes, were recently reviewed [15]. However, our study suggests that the cytotoxic impact of broccoli sprouts is more related to the presence of polyphenolic compounds, such as robinin and gentisic acid, than to sulforaphane (see Section 2.4, Table 2). This observation was confirmed by Shen et al. [16] who noted that robinin inhibited the growth of thyroid cancer cells (TPC-1 and SW1736) via apoptosis and caused the loss of membrane integrity in cells. This compound increased the activity of caspases 8 and 9 and the levels of Bax and caspase 3. Additionally, a decrease in the levels of Bcl-2, c-Myc, and cyclin-D1 was observed. In the case of gentisic acid, this is the first observation describing its role in the cytotoxic activity of broccoli sprout extract against thyroid cancer cells. Some previous observations indicated that gentisic acid was not cytotoxic to hepatoma tissue culture and a murine melanocyte cell line but significantly inhibited the proliferation and colony growth of C6 glioma cells [17].

It is known that different polyphenolic compounds may exhibit cytotoxic activity against thyroid cancer cells. Quercetin was shown to induce the differentiation of NIS in PTC cells, apigenin has been shown to increase iodine influx rate in PTC cells, and myricetin has been shown to enhance iodine retention and increase influx and decrease efflux via the Na^+^/I^−^ symporter in FTC cells. Natural plant compounds may also have an impact on MAPK, AKT/mTOR, and apoptosis in thyroid cancer. Curcumin, one of the most well-known curcuminoids, can change the expression of Bcl-2, Bcl-X, p53, p21, and PARP, which are involved in the apoptotic process, and subsequently inhibit the proliferation and invasion of human anaplastic thyroid cancer cells (SW-1736) [18,19].

### 2.2. Influence of Broccoli Sprout Extract on the Inflammation Process

**Figure 2:**
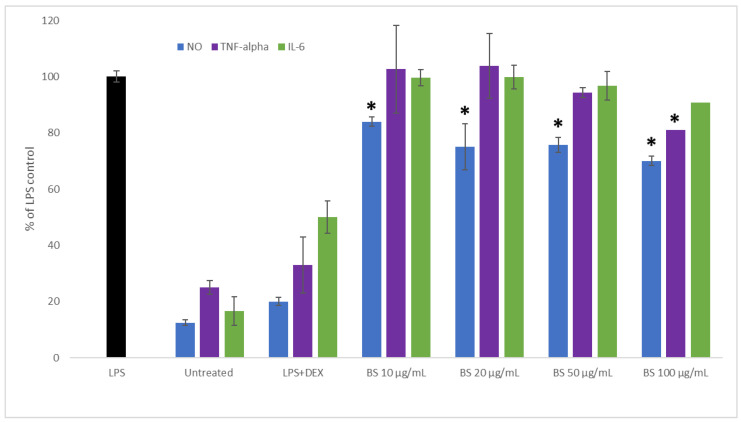
Effect of broccoli sprout extract (BS) on tumor necrosis factor-alpha (TNF-alpha), interleukin-6 (IL-6), and nitric oxide (NO) release in LPS-stimulated RAW 264.7 macrophages. RAW cells were pre-treated with 10, 20, 50, and 100 µg/mL of sprout extract for 1 h; afterwards, cells were incubated with (10 ng/mL) or without LPS (untreated) for the next 24 h. Dexamethasone (DEX) was used as a reference. Values are presented as the mean ± SD (standard deviation) of three independent experiments in triplicate. Statistical analyses were carried out using one-way ANOVA and Tukey’s post-hoc test with *: *p* < 0.05 against the LPS-stimulated cells.

Cancer is often accompanied by an inflammation process, which enables the progression of the disease. In addition, the chronic inflammation of normal tissue may increase the risk of developing many diseases, including carcinogenesis. This prompted us to determine the protective anti-inflammatory potential of broccoli sprout extract in an LPS-stimulated RAW 264.7 macrophage model. The results are presented in Figure 2. A significant inhibition of NO synthesis was observed in macrophages pre-treated with broccoli extract in all evaluated concentrations when compared to LPS-stimulated cells (*p* < 0.05). In the case of TNF-alpha, the evaluated broccoli sprout extract significantly decreased its release when compared to LPS-stimulated cells only in case of the highest concentration (100 µg/mL); the slight decrease observed at 50 µg/mL did not differ significantly from the LPS-treated cells. In our study, only a minor and insignificant suppression of IL-6 release was noted in LPS-stimulated macrophages pre-treated with broccoli sprout extract.

Brassica vegetables, such as broccoli, have significant anti-inflammatory properties [20]. Olszewska et al. [21] found that broccoli sprouts downregulated the release of pro-inflammatory cytokines (TNF-α and IL-6) from LPS-stimulated human peripheral blood mononuclear cells and increased the production of IL-10; furthermore, they suggested that phenolic constituents may also be responsible for the anti-inflammatory effects of broccoli sprouts and the stimulation of the secretion of IL-10. A similar decrease in NO and TNF-alpha release without an influence on IL-6 concentration was also observed in our previous study on kale sprouts [12].

### 2.3. Influence of Broccoli Sprout Extract on SOD1 Activity

**Figure 3:**
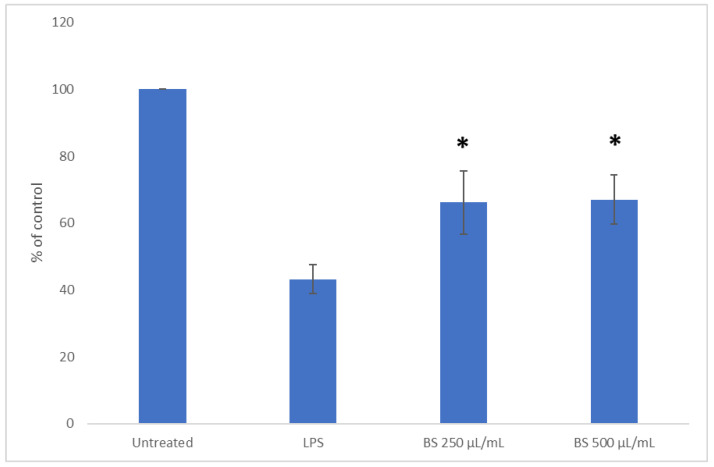
Effect of broccoli sprout extract (BS) on SOD1 activity in LPS-stimulated RAW 264.7 macrophages. RAW cells were pre-treated with 250 and 500 µg/mL of sprout extract for 1 h; afterwards, cells were incubated with (10 ng/mL) or without LPS (untreated) for the next 24 h. Values are presented as the mean ± SD (standard deviation) of three independent experiments in triplicate. Statistical analyses were carried out using one-way ANOVA and Tukey’s post-hoc test with *: *p* < 0.05 against the LPS-stimulated cells.

Antioxidant enzymes such as SOD play an important function in maintaining redox homeostasis and mediating the inflammation process. Thus, to obtain more thorough information on the anti-inflammatory potential of broccoli sprouts, we also decided to evaluate their influence on SOD activity in addition to their impact on pro-inflammatory mediators. The results (Figure 3) indicated that the activity of SOD in broccoli sprout extract-treated cells was significantly increased at the concentrations of 250 and 500 μg/mL in a dose-independent manner. Le et al. [22] indicated that the antioxidant activity of broccoli sprouts was associated with the presence of ascorbic acid, α-tocopherol, carotenoids, and phenolic compounds and that these compounds were responsible for 80−95% of the total antioxidant capacity in broccoli sprouts. The correlation weights (CWs) for the pairs of parameters based on the PCA model (Table 2) revealed that SOD activity was positively correlated with flavonoid robinin (CW = 0.126), which confirmed the abovementioned thesis.

The results of this study demonstrated that broccoli sprout extract was not able to restore the original antioxidant ability of cells by increasing SOD activity in LPS-stimulated macrophages. However, our previously published results indicated a considerable antioxidant activity (FRAP 50.67 ± 2.40 mmol Fe^2+^/100 g dw; DPPH 11.35 ± 0.44 mmol Trolox/100 g dw) and total phenolic compounds (4.26 ± 0.13 g/100 g dw) of the evaluated broccoli sprout extract [23].

It is known that the activities of antioxidant-related enzymes are downregulated in some antioxidant-treated cells because oxidative stress is directly attenuated by the antioxidants [24]. Kaiser et al. [3] noted that sulforaphane increased the levels of NQO1, TXNRD1, GSTM1, MGST1, SOD1, and PRDX1.

### 2.4. Chemometric Analysis of In Vitro Parameters

**Figure 4:**
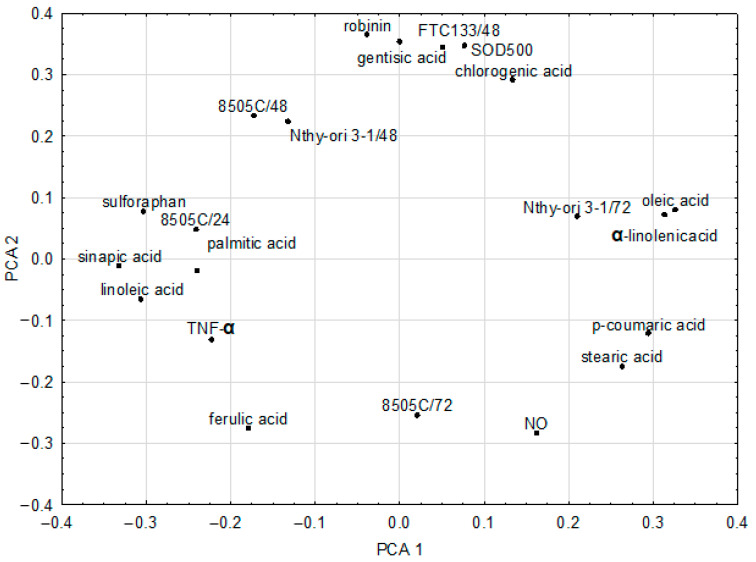
Loadings for the first two principal components of the PCA model (FTC133/48—48 h of incubation; 8505C/24/48—24 h or 48 h of incubation; Nthy-ori 3-1/48/72—48 h or 72 h of incubation; SOD500—SOD activity 500 μg/mL).

A statistically significant PCA model of two significant components was derived for the in vitro experiment. The parameters included in the PCA model were: NO, TNF-α, FTC133/48, 8505C/24, 8505C/48, 8505C/72, SOD500, Nthy-ori 3-1/48, Nthy-ori 3-1/72, sulforaphane, chlorogenic acid, p-coumaric acid, ferulic acid, gentisic acid, sinapic acid, oleic acid, linoleic acid, α-linolenic acid, stearic acid, palmitic acid, and robinin. Other parameters (IL-6, FTC133/24, FTC133/72, Nthy-ori 3-1/24, and SOD250) were not included in the model as they were considered noninformative. The model had two significant components and explained 75.3% of the variance in the original parameters, with eigenvalues of 2.47 and 2.05, respectively. The loadings for the first two principal components are shown in Figure 4. In our analysis, cytotoxic activity against thyroid cancer cells was not correlated with sulforaphane but was correlated with robinin and gentisic acid (Table 2).

### 2.5. Influence of Broccoli Sprouts on Rat Thyroid Histopathology

**Figure 5:**
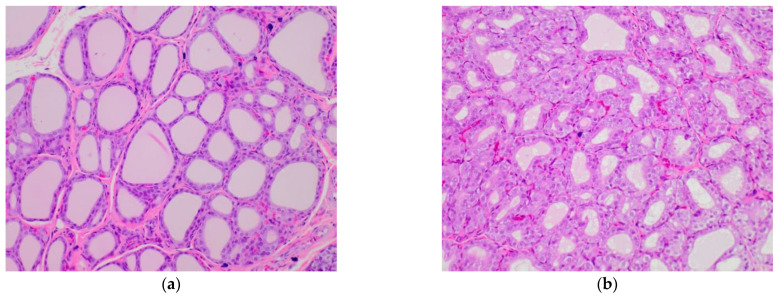

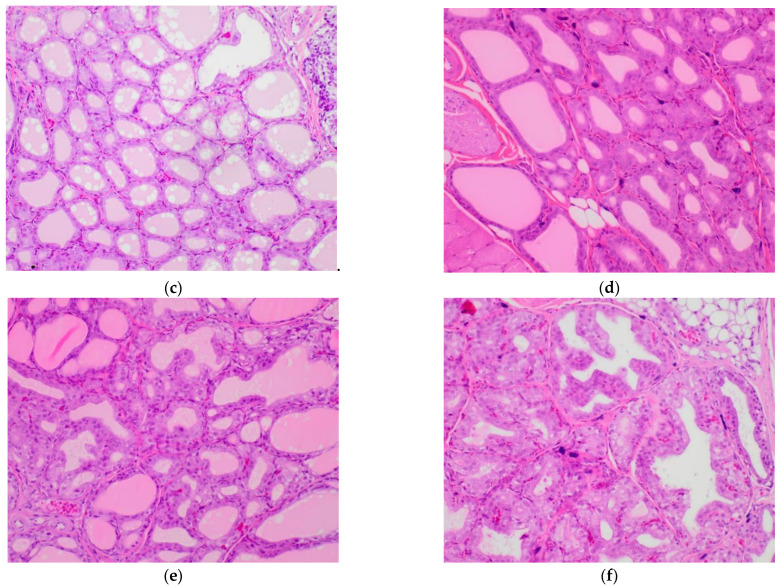
Histological appearance of the thyroid according to experimental group. All images were stained with hematoxylin–eosin at 200× magnification. (**a**) Group C: regular folliculi are lined by a rather flat epithelium and are full of colloid. (**b**) Group BS: folliculi are smaller with slightly higher cells. (**c**) Group DI: folliculi are also rather small and lined with cuboid cells, and the vacuolation of the colloid is prominent. (**d**) Group BS/DI: folliculi are smaller on the right and more dilated and lined by slightly flattened cells on the left. (**e**) Group S: variously sizes of folliculi, some with papillary projections. (**f**) Group BS/S: folliculi are smaller on the left and larger on the right; the papillary projections are evident.

As the final step of the experiment, the impact of broccoli sprouts on the thyroid was evaluated in healthy and hypothyroid rats. The histopathological analysis of thyroid glands was focused on the assessment of papillary formation, aggregates of lymphocytes, vacuole score, the quality of epithelial cells, follicular epithelial area, follicular luminal area, and overall thyroid area. The results for these qualitative and quantitative parameters are gathered in Table 3 and shown in Figure 5. The highest overall thyroid area in comparison to healthy rats was observed in the rats that received sulfadimethoxine (S), where the difference was significant. Broccoli sprouts did not have any significant influence on the follicular epithelial area, follicular luminal area, and overall thyroid area. However, a tendency for prevention against these negative increasing changes was mostly observed in the group of rats with iodide deficiency (BS/DI), while no such protective effect was noticed in rats that received sulfadimethoxine (BS/S). The same positive effect of broccoli sprouts was noted for the follicular luminal area of BS/DI rats. In healthy rats fed with broccoli sprouts (BS), 25% papillary formation was also observed. The highest papillary formation appeared in the S group and did not increase in the BS/S group; however, in the case of DI rats, the addition of broccoli sprouts to the diet increased papillary formation by 40%. The vacuolization process was observed in thyroid cells (40%) only in the DI group, without any aggregates of lymphocytes in any samples. In the control group, the thyroid follicles were lined by flattened epithelial cells; in the case of the DI and S groups, the follicles were lined by cuboidal cells in 60% and 100% of evaluated thyroids from each group, respectively. It should be noted that the addition of broccoli sprouts to the diet of healthy rats caused significant changes in the shape of the epithelial cells: 80% of the BS rats had cuboidal cells.

The morphological changes observed in rats fed with broccoli sprouts varied from those observed in rats receiving kohlrabi or rutabaga sprouts in the same models of hypothyroidism. In contrast to broccoli sprouts, kohlrabi sprouts caused the lining of follicles by cuboidal cells in 60% of the evaluated thyroids and rutabaga sprouts in only about 40% [4,25]. In the case of extensive follicle hypertrophy, broccoli sprouts induced these changes in heathy rats and rats with hypothyroidism, which is a similar observation to that reported for rats receiving rutabaga sprouts [25]. Kohlrabi sprouts induced this parameter only in a group of rats that received sulfadimethoxine as a harmful agent, without any effect observed in healthy animals [4]. Papillary formation was noted previously for a field mustard diet [26], with the highest development in rats fed with rape seeds. In all thyroid gland samples evaluated in this experiment, aggregates of lymphocytes were not detected, as was observed in rats receiving kohlrabi sprouts [4]. However, in the case of animals with iodine deficiency fed with rutabaga sprouts, lymphocyte aggregates were observed and coincided with high papillary formation [25].

We used the results obtained in our previous paper from a chemometric analysis (TSH, fT3, and fT4) [7] and combined them with the present histopathological study. The correlation weights for the pairs of parameters based on the PLS model, including histopathological parameters, are shown in Table 4.

The morphological changes observed in the rats being fed broccoli sprouts were positively correlated with TSH, which is one of the most significant factors influencing the morphofunctional status of follicles [27]. In particular, positive correlations were observed between TSH and follicular epithelial area (0.150), overall thyroid area (0.144), and papillary formation (0.128). Previous experiments on the influence of kohlrabi sprouts showed a negative correlation between fT3 and follicular epithelial area and fT3 and overall thyroid area [4]. Papillary formation occurring in the thyroid gland was positively corelated with follicular epithelial area and overall thyroid area, which was confirmed by Bell et al. in their study on mustard or rapeseed meal varieties with high glucosinolate content (oriental mustard (*Brassica juncea*), yellow sarson (*B. campestris*), and target (*B. napus*)) [26]. Rats fed with rutabaga sprouts and treated with sulfadimethoxine showed extreme papillary formation, which was significantly greater than that in the control group and the group receiving only sulfadimethoxine [25].

A previous study on the biochemical, hematological, and immunosignal parameters of rats with hypothyroidism receiving broccoli sprouts did not reveal a significant harmful effect [7]. The levels of TSH, fT3, and fT4 remained unchanged, which was suggested to be a protective effect against sulfadimethoxine-induced thyroid damage. Furthermore, thyroid thioredoxin reductase activity increased in response to the ingestion of sprouts. In animals with hypothyroidism, the sprouts were found to exert a beneficial influence on the antioxidant capacity of the thyroid gland. In comparison to the rats with iodine deficiency, the addition of broccoli sprouts to the diet decreased the level of IL-6. Despite the beneficial effect of broccoli sprouts, it should be noted that some negative histopathological changes were observed. It is possible that additional changes in histopathology would be visible and correlated with additional biochemical thyroid parameters in a longer experiment, which should be evaluated in the future.

### 2.6. Chemometric Analysis of In Vivo Parameters

**Figure 6:**
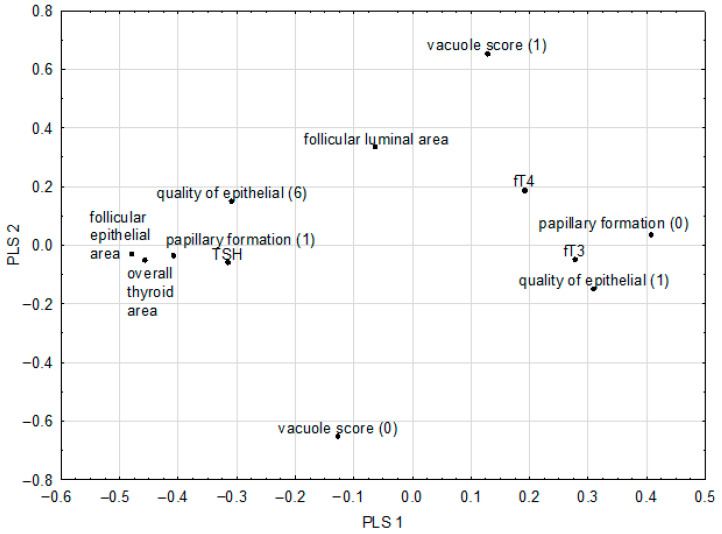
Loadings for the first two latent components of the PLS model (quality of epithelial cells: (1) flattened or (6) cuboidal; papillary formation: formation (1) or lack of papillary formation (0); vacuole score: observed (1) or lack of vacuole (0)).

A PLS model fulfilling the cross-validation criteria was constructed for the in vivo model. The following histopathological features were selected as predictive parameters: follicular epithelial area, follicular luminal area, overall thyroid area, papillary formation. In addition, the following dichotomous features were selected: vacuole score and quality of epithelial cells. fT3, fT4, and TSH were set as response parameters. The model had two significant components and explained 77.5% of the variance in the predictive parameters and 32.6% of the variance in the response parameters, with eigenvalues of 4.21 and 2.77, respectively. The loadings for the first two latent components are shown in Figure 6 and the correlation weights for the pairs of parameters based on the PLS model are shown in Table 4.

## 3. Materials and Methods

### 3.1. Plant Material

Four-day old broccoli sprouts were harvested by the Uniflora Company, Poland in 2018. A voucher specimen of broccoli seeds (*Brassica oleracea* L. convar. *botrytis* var. *cymosa*) was deposited at the Department of Food Chemistry and Nutrition, Jagiellonian University Medical College (No#BOCB/PP/PL 1036). Samples of raw sprouts were frozen at −20 °C for 48 h before the freeze-drying process, carried out as described previously [2].

### 3.2. Extract Preparation and Quantitative Analysis

The extract prepared and chemically profiled in our previous study [2] was used for the present experiments. Briefly, lyophilized broccoli sprouts were smashed in a mortar for over 1.5 min and then incubated (forced-air oven at 30–40 °C) for 4 h to promote the hydrolysis of glucoraphanin to sulforaphane by myrosinase [28]. The obtained material was extracted with methanol using a Soxhlet apparatus. The methanol broccoli sprout extract (BSE) was further evaporated, and the dry residues were dissolved in DMSO for the evaluation of the cytotoxic and anti-inflammatory activity of broccoli sprouts.

Sulforaphane (113.33 ± 12.58 mg/100 g dw) concentration in the BSE was evaluated previously via the UPLC–MS/MS method. An HPLC analysis of BSE showed several phenolic acids and flavonoids, including chlorogenic acid (37.26 ± 0.6 mg/100 g dw); p-coumaric acid (27.75 ± 0.70 mg/100 g dw); ferulic acid (73.85 ± 3.50 mg/100 g dw); gentisic acid (80.80 ± 4.79 mg/100 g dw); sinapic acid (140.53 ± 3.17 mg/100 g dw); and robinin (1.04 ± 0.10 mg/100 g dw). A GC analysis of the fatty acids profile showed oleic (45.5%), linoleic (20.8%), alpha-linolenic (17.06%), palmitic (5.7%), and stearic (2.8%) acids [2].

### 3.3. Broccoli Sprouts

#### 3.3.1. Cytotoxic Activity of Broccoli Sprouts

Cytotoxic activity was tested on the following human thyroid cancer and normal cells: (1) follicular thyroid carcinoma, derived from lymph node metastasis FTC-133, ECACC 94060901; (2) undifferentiated thyroid carcinoma 8505C, ECACC 94090184; and (3) thyroid follicular epithelial cells Nthy-ori 3-1, ECACC 90011609. The cells were grown in standard conditions (37 °C, 5% CO_2_, relative humidity) and culture media (DMEM/F12 for FTC133 and 8505C and RPMI1640 for Nthy-ori 3-1) supplemented with 10% fetal bovine serum (FBS) and 1% antibiotic solution (10,000 U penicillin and 10 mg streptomycin/mL). The examined extracts were diluted in the culture media from freshly made stock solution in DMSO (10 mg/mL) to the working concentrations (from 0 to 300 μg/mL). Cell viability was determined using an LDH assay as described previously [29]. Briefly, cells were seeded onto 96-well plates (1.5 × 10^4^ cells/well) and cultured for 24 h. Then, the culture medium was replaced with the same medium containing different concentrations (0.025–0.3 mg/mL) of the broccoli sprout extract. After 24, 48, and 72 h of incubation, cell viability was determined. The absorbance was measured at 490 nm using a BioTek Synergy microplate reader (BioTek Instruments Inc., Winooski, VT, USA). All analyses were performed in triplicate; the results are expressed as % of cell viability (mean ± SD) and IC_50_ values (concentration at which the viability was inhibited by 50 percent).

#### 3.3.2. Inflammation Model

For the anti-inflammatory assays, RAW 264.7 murine macrophages were seeded onto 96-well plates (1.5 × 10^5^ cells/well) and pre-treated with the tested broccoli sprout extract (10–100 µg/mL) for 1 h, followed by the addition of 10 ng/mL of LPS to induce the inflammation process, according to Paśko et al. [30]. Dexamethasone (0.5 µg/mL) was used as a reference drug. The incubation was continued for the next 24 h. Cell culture supernatants were used for further analysis.

#### 3.3.3. Nitric Oxide Determination

A Griess reagent kit was obtained from Promega Corporation (Madison, Winooski, VT, USA) and a nitric oxide evaluation was performed according to the manufacturer’s instructions. The analysis was performed in RAW 264.7 cell culture supernatants on three replicates using a BioTek Synergy microplate reader (BioTek Instruments Inc., Winooski, VT, USA); the results are shown as % of LPS control.

#### 3.3.4. TNF-Alpha and IL-6 Analysis

Cytokine determination was performed with human ELISA kits (Bioassay Technology Laboratory, Shanghai, China) according to the manufacturer’s instructions. RAW 264.7 cell culture supernatants (see Section 3.3.3) were used for the analysis, which was performed for three replicates using a microplate reader (BioTek Instruments Inc., Winooski, VT, USA); the results are shown as % of LPS control.

#### 3.3.5. Human Superoxide Dismutase 1 Activity

An evaluation of human superoxide dismutase (Cu-Zn) activity was performed with human ELISA kits (Bioassay Technology Laboratory, Shanghai, China) according to the manufacturer’s instructions. RAW 264.7 cell culture supernatants (see Section 3.3.3) were used for the analysis, which was performed for three replicates using a microplate reader (BioTek Instruments Inc., Winooski, VT, USA); the results are shown as % of control (untreated).

### 3.4. Broccoli Sprouts—Experiment In Vivo

#### 3.4.1. Animals

Detailed information about the animal experiment was described previously [7]. Seventy-two 4-week-old Wistar male rats (mean weight 249.5 ± 9.1 g) were maintained in the Animal House of the Faculty of Pharmacy, Jagiellonian University Medical College. After the acclimatization process, the rats were divided into 6 groups, each consisting of 12 animals, and fed one of the following diets: a standard diet (C); an iodine deficient diet (DI); a broccoli sprout diet (BS); an iodine deficient diet with broccoli sprouts (BS/DI); a standard diet with sulfadimethoxine delivered in drinking water (S); or a diet containing broccoli sprouts and sulfadimethoxine (BS/S). The dietary models described above were applied to imitate the healthy status of the thyroid (standard diet) or hypothyroidism induced by diet with iodine deficiency causing thyroid hyperplasia or sulfadimethoxine-impaired thyroid hormone synthesis [4]. The protocols for animal experiments were approved by the Animal Experimentation Committee of Jagiellonian University, Kraków, Poland (No. 76/2014).

#### 3.4.2. Histopathology of Rat Thyroids

The thyroid histopathology analysis was described in detail previously by Paśko et al. [4].

The assessment of histology was completed in a blinded manner. Both qualitative description and image acquisition were performed with an Axioskop microscope (Zeiss GmbH, Oberkochen, Germany). All the sections containing recognizable thyroid tissue were photographed using a Plan NeoFluar 2.5× lens and a Nikon D5300 camera. For calibrating the images, a 1 mm microscopic grid (Zeiss, Jena, Germany) was photographed with the same microscopy system. The images were transferred to standard personal computer and processed interactively using the Photoshop CS4 (Adobe Systems Inc., San Jose, CA, USA) software. Measurements were made with the AnalySIS 3.2 pro (Soft Imaging Systems GmbH, Münster, Germany) image analysis system controlled by a program written by one of the authors (K.O.) in the Imaging C macro language. The program loaded the successive image files and recorded the surface area of the thyroid tissue as well as area of the empty ‘holes’ that constituted the lumens of thyroid follicles.

### 3.5. Statistical Analysis

All experiments were performed in triplicate, and the data were reported as the mean ± standard deviation (SD). The results obtained for subsequent groups were analyzed using a one-way analysis of variance (ANOVA) followed by a post hoc Tukey’s test. The differences among the groups were considered to be statistically significant when the *p* values were 0.05 or less. This part of the statistical analysis was performed using STATISTICA v. 13.3. (TIBCO Software Inc., Palo Alto, CA, USA).

#### Chemometric Methods

The partial least square approach (PLS) and principal component analysis (PCA) were applied to describe the correlation structure between the parameters in the in vivo and in vitro models, respectively. The details of our implementation of both models for were described in several previous papers [7,31,32,33]. Statistical analyses were performed using the SIMCA-P v.9 (Umetrics, Umeå, Sweden) package. The STATISTICA v. 13.3. (TIBCO Software Inc., Palo Alto, CA, USA) software was used for the visualization of results. The correlation weights in both models were calculated using software delivered by MP System Co. (Kraków, Poland).

## 4. Conclusions

The determination of the safety profile of broccoli sprouts, a popular element of our daily diet, is crucial especially for people with thyroid dysfunctions. Although the health effects of these sprouts have been well known for years, the inconclusive results of limited human and animal studies did not clearly indicate their impact on thyroid function. The results of our study indicate an interesting potential of broccoli sprouts to decrease the viability of thyroid cancer cells and to prevent the associated inflammation. Moreover, our results suggest a satisfactory safety profile of the sprouts both in vitro and in vivo; however, a further in-depth evaluation of this problem is still needed.

Information on the influence of brassica vegetables on thyroid function is of great importance in terms of public health, especially when taking into account that that risk of iodine deficiency and hypothyroidism in the global population is still increasing, and thyroid cancer will replace colorectal cancer as the fourth leading cancer diagnosis by 2030.

## Figures and Tables

**Table 1:** Cytotoxic activity of broccoli sprout extract (BSE) against normal (Nthy-ori 3-1) and cancer (FTC-133 and 8505C) thyroid cells after 24, 48, and 72 h incubation expressed as IC_50_ values (µg/mL).

IC_50_ µg/mL	24 h	48 h	72 h
Nthy-ori 3-1	>C_max_	>C_max_	>C_max_
FTC-133	35.2	24.7	16.9
8505C	114.1	57.5	29.5

**Table 2:** Correlation weights for the pairs of parameters based on PCA model.

Pairs of Correlated Parameters		Correlation Weights
SOD500	robinin	0.126
FTC133/48	robinin	0.126
FTC133/48	gentisic acid	0.120
FTC133/48	SOD500	0.119

FTC133/48–48 h of incubation; SOD500–SOD activity 500 μg/mL.

**Table 3:** Qualitative and quantitative parameters describing the changes observed during the histopathological examination of thyroid glands from the investigated rats (see Section 3.4.1 for an explanation of the groups’ acronyms).

Parameters	C	BS	DI	BS/DI	S	BS/S
Papillary formation (%)	0%	25%	0%	40%	60%	60%
Aggregates of lymphocytes (%)	0%	0%	0%	0%	0%	0%
Vacuolization of the colloid (%)	0%	0%	40%	0%	0%	0%
Shape of epithelial cells (flattened vs. cuboidal) (%)	100% Flattened	80% Cuboidal	60% Cuboidal	40% Cuboidal	100% Cuboidal	80% Cuboidal
Follicular epithelial area (mm^2^)	1.57 ± 0.14 ^ab^	1.62 ± 0.82 ^c^	3.06 ± 0.71	2.36 ± 0.55 ^de^	4.37 ± 1.03 ^bcd^	3.96 ± 0.65 ^ae^
Follicular luminal area (mm^2^)	0.32 ± 0.06 ^a^	0.43 ± 0.13	0.69 ± 0.24 ^abc^	0.31 ± 0.04 ^c^	0.41 ± 018	0.45 ± 0.06 ^b^
Overall thyroid area (mm^2^)	1.79 ± 0.25 ^a^	1.61 ± 0.56	3.50 ± 1.13	2.67 ± 0.60 ^b^	4.64 ± 1.40 ^ab^	4.42 ± 0.63

(%)—percent of animals in which changes were observed. Results marked with the same letters within each row differ significantly (*p* < 0.05).

**Table 4:** Correlation weights for the pairs of parameters based on the PLS model.

Pairs of Correlated Parameters		Correlation Weights
Overall thyroid area	Follicular epithelial area	0.219
Follicular luminal area	Vacuolization of the colloid	0.218
Follicular epithelial area	Papillary formation	0.196
Overall thyroid area	Papillary formation	0.186
Follicular epithelial area	TSH	0.150
Overall thyroid area	TSH	0.144
Cuboidal epithelial shape	Follicular luminal area	0.129
Papillary formation	TSH	0.128
Overall thyroid area	Flattened epithelial shape	−0.133
Follicular epithelial area	Flattened epithelial shape	−0.137
Overall thyroid area	Lack of papillary formation	−0.183
Follicular epithelial area	Lack of papillary formation	−0.194
Follicular luminal area	Lack of vacuolization of the colloid	−0.202

## Data Availability

Data available upon request.

## References

[1] Fahey J.W., Kensler T.W. (2021). The challenges of designing and implementing clinical trials with broccoli sprouts… and turning evidence into public health action. Front. Nutr..

[2] Paśko P., Tyszka-Czochara M., Galanty A., Gdula-Argasińska J., Żmudzki P., Bartoń H., Zagrodzki P., Gorinstein S. (2018). Comparative study of predominant phytochemical compounds and proapoptotic potential of broccoli sprouts and florets. Plant Foods Hum. Nutr..

[3] Kaiser A.E., Baniasadi M., Giansiracusa D., Giansiracusa M., Garcia M., Fryda Z., Wong L.T., Bishayee A. (2021). Sulforaphane: A broccoli bioactive phytocompound with cancer preventive potential. Cancers.

[4] Paśko P., Okoń K., Prochownik E., Krośniak M., Francik R., Kryczyk-Kozioł J., Grudzińska M., Tyszka-Czochara M., Malinowski M., Sikora. J. (2022). The Impact of Kohlrabi Sprouts on Various Thyroid Parameters in Iodine Deficiency-and Sulfadimethoxine-Induced Hypothyroid Rats. Nutrients.

[5] Di Dalmazi G., Giuliani C. (2021). Plant constituents and thyroid: A revision of the main phytochemicals that interfere with thyroid function. Food Chem. Toxicol..

[6] Langer P., Michajlovskij N., Sedlak J., Kutka M. (1971). Studies on the antithyroid activity of naturally occurring L-5-vinyl-2-thiooxazolidone in man. Endokrinologie.

[7] Paśko P., Krośniak M., Prochownik E., Tyszka-Czochara M., Fołta M., Francik R., Sikora J., Malinowski M., Zagrodzki P. (2018). Effect of broccoli sprouts on thyroid function, haematological, biochemical, and immunological parameters in rats with thyroid imbalance. Biomed. Pharmacother..

[8] Dobrowolska-Iwanek J., Zagrodzki P., Prochownik E., Jarkiewicz A., Paśko P. (2019). Influence of brassica sprouts on short chain fatty acids concentration in stools of rats with thyroid dysfunction. Acta Pol. Pharm..

[9] Shapiro T.A., Fahey J.W., Dinkova-Kostova A.T., Holtzclaw W.D., Stephenson K.K., Wade K.L., Ye L., Talalay P. (2006). Safety, tolerance, and metabolism of broccoli sprout glucosinolates and isothiocyanates: A clinical phase I study. Nutr. Cancer.

[10] Chartoumpekis D.V., Ziros P.G., Chen J.G., Groopman J.D., Kensler T.W., Sykiotis G.P. (2019). Broccoli sprout beverage is safe for thyroid hormonal and autoimmune status: Results of a 12-week randomized trial. Food Chem. Toxicol..

[11] Galanty A., Zagrodzki P., Gdula-Argasińska J., Grabowska K., Koczurkiewicz-Adamczyk P., Wróbel-Biedrawa D., Podolak I., Pękala E., Paśko P. (2021). A Comparative survey of anti-melanoma and anti-inflammatory potential of usnic acid enantiomers—A Comprehensive in vitro approach. Pharmaceuticals.

[12] Paśko P., Galanty A., Zagrodzki P., Żmudzki P., Bieniek U., Prochownik E., Domínguez-Álvarez E., Bierła K., Łobiński R., Szpunar J. (2022). Varied effect of fortification of kale sprouts with novel organic selenium compounds on the synthesis of sulphur and phenolic compounds in relation to cytotoxic, antioxidant and anti-inflammatory activity. Microchem. J..

[13] Wang L., Tian Z., Yang Q., Li H., Guan H., Shi B., Hou P., Ji M. (2015). Sulforaphane inhibits thyroid cancer cell growth and invasiveness through the reactive oxygen species-dependent pathway. Oncotarget.

[14] Chatterjee S., Rhee Y., Chung P.S., Ge R.F., Ahn J.C. (2018). Sulforaphene enhances the efficacy of photodynamic therapy in anaplastic thyroid cancer through Ras/RAF/MEK/ERK pathway suppression. J. Photochem. Photobiol. B..

[15] Kamal M.M., Akter S., Lin C.N., Nazzal S. (2020). Sulforaphane as an anticancer molecule: Mechanisms of action, synergistic effects, enhancement of drug safety, and delivery systems. Arch. Pharmacal Res..

[16] Shen Y., Velu P., Huang X., Dang T. (2022). Evaluation of apoptotic and cytotoxic effect of robinin in TPC-1 and SW1736 human thyroid cancer cells. Phcog. Mag..

[17] Abedi F., Razavi B.M., Hosseinzadeh H. (2020). A review on gentisic acid as a plant derived phenolic acid and metabolite of aspirin: Comprehensive pharmacology, toxicology, and some pharmaceutical aspects. Phytother. Res..

[18] Sharifi-Rad J., Rajabi S., Martorell M., López M.D., Toro M.T., Barollo S., Pezzani R. (2020). Plant natural products with anti-thyroid cancer activity. Fitoterapia.

[19] Varnamkhasti K., Tavakoli P., Rouhi L., Raisi S. (2021). Cytotoxicity, Apoptosis induction and change of p53, PARP, p21 and Bcl-2 genes expression in the human anaplastic thyroid carcinoma cells line (SW-1736) with curcumin. Genet. Appl..

[20] Tilg H. (2015). Cruciferous vegetables: Prototypic anti-inflammatory food components. Clin. Phytosci..

[21] Olszewska M.A., Granica S., Kolodziejczyk-Czepas J., Magiera A., Czerwińska M.E., Nowak P., Rutkowska M., Wasiński P., Owczarek A. (2020). Variability of sinapic acid derivatives during germination and their contribution to antioxidant and anti-inflammatory effects of broccoli sprouts on human plasma and human peripheral blood mononuclear cells. Food Funct..

[22] Le T.N., Chiu C.H., Hsieh P.C. (2020). Bioactive compounds and bioactivities of Brassica oleracea L. var. italica sprouts and microgreens: An updated overview from a nutraceutical perspective. Plants.

[23] Paśko P., Prochownik E., Galanty A., Bartoń H., Tyszka-Czochara M., Zagrodzki P., Zachwieja Z. (2018). Comparative study of antioxidant capacity and total polyphenols content of broccoli sprouts and florets. Bromat. Chem. Toksykol..

[24] Chou S.T., Peng H.Y., Hsu J.C., Lin C.C., Shih Y. (2013). Achillea millefolium L. essential oil inhibits LPS-induced oxidative stress and nitric oxide production in RAW 264.7 macrophages. Int. J. Mol. Sci..

[25] Paśko P., Okoń K., Krośniak M., Prochownik E., Żmudzki P., Kryczyk-Kozioł J., Zagrodzki P. (2018). Interaction between iodine and glucosinolates in rutabaga sprouts and selected biomarkers of thyroid function in male rats. J. Trace Elem. Med. Biol..

[26] Bell J.M., Benjamin B.R., Giovannetti P.M. (1972). Histopathology of thyroids and livers of rats and mice fed diets containing Brassica glucosinolates. Can. J. Anim. Sci..

[27] Rajab N.M.A., Ukropina M., Cakic-Milosevic M. (2017). Histological and ultrastructural alterations of rat thyroid gland after short-term treatment with high doses of thyroid hormones. Saudi J. Biol. Sci..

[28] De Groot A.P., Willems M.I., De Vos R.H. (1991). Effects of high levels of Brussels sprouts in the diet of rats. Food Chem. Toxicol..

[29] Galanty A., Popiół J., Paczkowska-Walendowska M., Studzińska-Sroka E., Paśko P., Cielecka-Piontek J., Pękala E., Podolak I. (2021). (+)-Usnic acid as a promising candidate for a safe and stable topical photoprotective agent. Molecules.

[30] Paśko P., Galanty A., Zagrodzki P., Luksirikul P., Barasch D., Nemirovski A., Gorinstein S. (2021). Dragon fruits as a reservoir of natural polyphenolics with chemopreventive properties. Molecules.

[31] Paśko P., Prochownik E., Krośniak M., Tyszka-Czochara M., Francik R., Marcinkowska M., Sikora J., Malinowski M., Zagrodzki P. (2020). Animals in iodine deficiency or sulfadimethoxine models of thyroid damage are differently affected by the consumption of Brassica sprouts. Biol. Trace Elem. Res..

[32] Pasko P., Gdula-Argasinska J., Podporska-Carroll J., Quilty B., Wietecha-Posluszny R., Tyszka-Czochara M., Zagrodzki P. (2015). Influence of selenium supplementation on fatty acids profile and biological activity of four edible amaranth sprouts as new kind of functional food. J. Food Sci. Technol..

[33] Zagrodzki P., Paśko P., Galanty A., Tyszka-Czochara M., Wietecha-Posłuszny R., Rubió P.S., Bartoń H., Prochownik E., Muszyńska B., Sułkowska-Ziaja K. (2020). Does selenium fortification of kale and kohlrabi sprouts change significantly their biochemical and cytotoxic properties?. J. Trace Elem. Med. Biol..

